# APOE4 carriers across neurodegenerative diseases have common systemic biological dysfunction

**DOI:** 10.1002/alz70855_099496

**Published:** 2025-12-23

**Authors:** Artur Shvetcov, Erik C.B. Johnson, Laura M Winchester, Keenan A. Walker, Heather M Wilkins, Jeffrey M. Burns, Russell H Swerdlow, Chad M Slawson, Caitlin A Finney, Global Neurodegeneration Proteomics Consortium

**Affiliations:** ^1^ Discipline of Psychiatry and Mental Health, University of New South Wales, Sydney, NSW, Australia; ^2^ Translational Dementia Research Group, Westmead Institute for Medical Research, Westmead, NSW, Australia; ^3^ Department of Psychological Medicine, Sydney Children's Hospitals Network, Sydney, NSW, Australia; ^4^ Department of Neurology, Emory University School of Medicine, Atlanta, GA, USA; ^5^ Goizueta Alzheimer's Disease Research Center, Emory University, Atlanta, GA, USA; ^6^ Department of Psychiatry, University of Oxford, Oxford, United Kingdom; ^7^ Laboratory of Behavioral Neuroscience, National Institute on Aging, Intramural Research Program, Baltimore, MD, USA; ^8^ University of Kansas Alzheimer's Disease Research Center, Kansas City, KS, USA; ^9^ Department of Neurology, University of Kansas School of Medicine, Kansas City, KS, USA; ^10^ Department of Biochemistry and Molecular Biology, University of Kansas Medical Center, Kansas City, KS, USA; ^11^ University of Kansas Alzheimer's Disease Research Center, Fairway, KS, USA; ^12^ Department of Cell Biology and Physiology, University of Kansas Medical Center, Kansas City, KS, USA; ^13^ University of Kansas Cancer Center, University of Kansas Medical Center, Kansas City, KS, USA; ^14^ School of Medical Sciences, The University of Sydney, Sydney, NSW, Australia

## Abstract

**Background:**

Apolipoprotein E ε4 (APOE4) is the biggest genetic risk factor for sporadic Alzheimer's disease (AD). There is also evidence that APOE4 carriers have an increased risk of and/or altered disease progression for other age‐associated neurodegenerative diseases, including frontotemporal dementia (FTD) and Parkinson's disease (PD). Despite this, little is known about the unifying mechanisms underlying APOE4 carriers’ vulnerability to these diseases.

**Methods:**

To identify APOE4‐specific mechanisms, we performed machine learning‐based proteome profiling across the plasma, cerebrospinal fluid (CSF), and brains of APOE4 carriers and non‐carriers with no‐impairment (NI), AD, FTD, and PD. Genetic, SomaScan 7k assay proteomic, and clinical data was sourced from the Global Neurodegeneration Proteomics Consortium for 5057 NI, 2061 AD, 147 FTD, and 897 PD patients. We obtained genetic and label free mass spectrometry proteomic data for the dorsolateral prefrontal cortex (dlPFC) of 47 NI, 49 AD, 31 FTD, and 33 PD patients from the Accelerating Medicines Partnership for AD UPenn Proteomics Study. APOE4‐associated proteins were identified using mutual information and classification and regression tree modelling followed by functional network analyses. A correlation network analysis was used to elucidate the relationship between proteins and clinical variables.

**Results:**

Compared to non‐carriers, all APOE4 carriers irrespective of diagnosis had the same proteome signature across the plasma and CSF. These proteins were associated with immune dysregulation, inflammation, protein folding and phosphorylation, and DNA/RNA processes. dlPFC proteome profiling confirmed that the same biological pathways were altered in APOE4 carriers. There were, however, disease‐specific relationships between APOE4‐associated proteins and clinical variables. In AD, APOE4‐associated proteins were related to alcohol use and smoking. In FTD, these were related to diabetes, hypertension, and depression. In PD, APOE4‐assocaited proteins were related to age, alcohol, congestive heart failure, hyperlipidemia, and hypertension.

**Conclusion:**

Our findings demonstrate that APOE4 carriage defines altered biological pathways across the plasma, CSF, and brains irrespective of diagnosis, suggesting that APOE4 carriers have systemic biological changes beyond the central nervous system. Further, APOE4 carriers’ underlying biological state may be essential but not sufficient for developing neurodegenerative diseases. Lifestyle and clinical factors appear to interact with the APOE4 proteomic signature in a disease‐specific manner.

